# Structural features of reconstituted wheat wax films

**DOI:** 10.1098/rsif.2016.0396

**Published:** 2016-07

**Authors:** Elias Pambou, Zongyi Li, Mario Campana, Arwel Hughes, Luke Clifton, Philipp Gutfreund, Jill Foundling, Gordon Bell, Jian R. Lu

**Affiliations:** 1 Biological Physics Group, School of Physics and Astronomy, University of Manchester, Oxford Road, Manchester M13 9PL, UK; 2 STFC ISIS Facility, Rutherford Appleton Laboratory, Didcot OX11 0QX, UK; 3 Institut Laue-Langevin, 71 avenue des Martyrs, 38000 Grenoble, France; 4 Syngenta, Jealott's Hill International Research Centre, Bracknell, Berkshire RG42 6EY, UK

**Keywords:** wheat, *Triticum*, plant waxes, cuticular films, neutron reflection

## Abstract

Cuticular waxes are essential for the well-being of all plants, from controlling the transport of water and nutrients across the plant surface to protecting them against external environmental attacks. Despite their significance, our current understanding regarding the structure and function of the wax film is limited. In this work, we have formed representative reconstituted wax film models of controlled thicknesses that facilitated an *ex vivo* study of plant cuticular wax film properties by neutron reflection (NR). *Triticum aestivum* L. (wheat) waxes were extracted from two different wheat straw samples, using two distinct extraction methods. Waxes extracted from harvested field-grown wheat straw using supercritical CO_2_ are compared with waxes extracted from laboratory-grown wheat straw via wax dissolution by chloroform rinsing. Wax films were produced by spin-coating the two extracts onto silicon substrates. Atomic force microscopy and cryo-scanning electron microscopy imaging revealed that the two reconstituted wax film models are ultrathin and porous with characteristic nanoscale extrusions on the outer surface, mimicking the structure of epicuticular waxes found upon adaxial wheat leaf surfaces. On the basis of solid–liquid and solid–air NR and ellipsometric measurements, these wax films could be modelled into two representative layers, with the diffuse underlying layer fitted with thicknesses ranging from approximately 65 to 70 Å, whereas the surface extrusion region reached heights exceeding 200 Å. Moisture-controlled NR measurements indicated that water penetrated extensively into the wax films measured under saturated humidity and under water, causing them to hydrate and swell significantly. These studies have thus provided a useful structural basis that underlies the function of the epicuticular waxes in controlling the water transport of crops.

## 1. Introduction

Plant surfaces are covered by a waxy cuticle that is primarily composed of an outer cuticular wax film and an inner polymeric cutin, with the cuticular wax often well embedded within the extracellular cutin matrix [1]. The wax layer forms a thin skin of thickness ranging from approximately 10 nm to 10 µm, depending on species—often with crystalline structures embedded on the outer surface—providing a barrier between the external environment and the underlying cells and vascular tissue found within the leaf or stem [1,2]. The wax layer plays an important role in moderating the transport, uptake and loss of nutrients and water. It also protects the plant from fungi and other pathogens. The deposition and retention of spray chemicals occurs on this surface, which must be crossed in order to get to the underlying tissue [3–6].

In order to better determine the role that the waxy cuticle plays in plant maintenance, wetting and nutrient transport, it is essential to establish the physical structure and chemical nature of the wax film [3]. The complex chemical composition of the lipid mixture that makes up cuticular waxes has in the past been determined for a number of plant leaves using—but not limited to—NMR spectroscopy, differential scanning calorimetry (DSC) and gas chromatography [7,8]. The average composition of wheat waxes was analysed by Koch *et al.* [9] using these techniques. While just 89% of the total mass could be accounted for, waxes were found to consist primarily of *n*-alkanols (C_22–30_), esters (C_44_, 12 m%) and alkanes (C_27–33_) with the *n*-alkanol C_28_ being the most prominent (66 m%) [6,10,11].

The physical and chemical composition of plant waxes has a significant influence upon wax crystallinity, transport properties and their interaction with adjuvants and surfactants; and therefore represents an important area of basic and applied research. The fundamental role of the cuticular wax ‘skin’ is to act as a barrier to the diffusive passage of water and nutrients across the plant interface [7,11]. Stripping the cuticular wax layer from plants using organic solvents, or otherwise, leads to an increase in water permeability of up to four orders of magnitude, emphasizing the importance of the cuticular wax's role in plant maintenance [12,13]. Furthermore, studies into water transport across the cuticular waxes of various crop species have shown water permeability through the cuticular wax to be independent of both cuticle thickness and coverage [5,14]. Crops such as maize for example are known to be extremely proficient at water retention despite a comparatively thin layer of wax and being predominantly grown in hot and arid climates. Plant cuticular waxes have also been seen to show significantly better barrier properties than cutin mimic polymer films of comparable thicknesses, indicating the complexity and importance of the cuticular waxes to the survival and success of individual plants [5].

A key target in adjuvant and plant science is to characterize the wax structure and transport properties within the cuticle. This will provide us with an understanding of the influence of the environment and wax composition upon the plant's interaction with adjuvants as they are deposited on a leaf surface. A representative model of a wax cuticular film would contribute to the understanding of the role the outermost leaf barrier plays.

Neutron reflectivity has become a widely used technique over the last two decades in the field of surface chemistry and physics. It has allowed the soft matter community to investigate the structural properties of adsorbed surfactants, lipids or proteins and now it is being used to achieve a better structural determination of cuticular wax films formed upon the surface of plants [15–18]. The combination of neutron reflection (NR) with complimentary techniques such as spectroscopic ellipsometry (SE), atomic force microscopy (AFM) and cryo-SEM (scanning electron microscopy) can yield additional information regarding possible structural or density variations within the wax films, or regarding the extrusions and patterning effects found on the surface; all of which may have a strong influence on the film's properties. The advancement of high-resolution imaging techniques over recent years has been crucial to the study as they enable direct comparisons to be made regarding different plant species. The successful fitting and interpretation of the neutron reflectivity profiles could pave the way for the investigation of more complex non-homogeneous structures found in nature.

## 2. Neutron reflection: technique and analysis

### 2.1. Theory

NR can be used to study the nanoscale structure and composition of a material adsorbed onto a surface or interface [17]. For specular reflection, a highly collimated beam of neutrons is elastically scattered off an optically flat surface at a given incidence angle into a detector. The reflected intensity of neutrons is measured as a function of the momentum transfer, *Q*, [=(4*π* sin*θ*)/*λ*], where *λ* is the incident neutron wavelength and *θ* is the incident angle. Analogous to the specular reflectivity of light, wavelength can be related to the neutron refractive index (*n*) of non-absorbent materials, defined by *n* *=* 1 − (*λ*^2^*ρ*)/2*π*, where *ρ* is the scattering length density (SLD) of the material. The total SLD of a molecule, film or individual layer is an additive quantity, dependent on the coherent scattering lengths and volume fraction of each isotopic component which makes up the region in question [19,20]. Commonly used SLD values are listed in table 1.

**Table 1.** Scattering length densities *ρ*, for commonly used materials in neutron reflectometry.

material	*ρ* (Å^−2^)
H_2_O	−0.56×10^−6^
D_2_O	6.35×10^−6^
silicon (Si)	2.07×10^−6^
silicon oxide (SiO_2_)	3.49×10^−6^
air	0
wheat waxes [9]	−0.29×10^−6^

The neutron SLD of a material studied can therefore be determined if its molecular components and chemical composition are known. The density of the wheat wax films was determined using the crude assumption that it is primarily made up of 66 m% C_28_ alcohol chains and 12 m% C_44_ ester (89% of extracted wax compound was identified; minor components, e.g. C_22–30_ alcohols, fatty acids and alkanes have very similar scattering lengths and can be ignored), as reported by Koch; giving an SLD value of −0.29 × 10^−6^ Å^−2^.

Because neutron scattering length varies between elemental isotopes, a technique referred to as isotopic substitution can be exploited to change the reflectivity of a material without significantly altering its chemical properties. Isotopic substitution can be used to change both the neutron SLD and scattering contribution of a given material or component by varying its scattering contrast with respect to the bulk and support substrate. A common example of this practice is the isotopic substitution of water. Combinations of H_2_O and D_2_O, which have very different SLDs (table 1), can be used to create a wide range of bulk solutions of defined SLD to help highlight otherwise buried interfacial features and describe structural details in different ways. Water contrast matched to silicon (CMSi) in combination with a Si substrate is often used in NR; made up of 38.1% D_2_O and 61.9% H_2_O to give an overall SLD of ‘2.07’. Under this contrast and ignoring the small reflectivity contribution from the thin oxide layer, the observed specular reflectivity profile is caused solely by the interfacial material, allowing it to be studied exclusively.

As SLD varies with isotopic composition, the data-fitting of a reflectivity profile measured under multiple isotopic contrasts to a single structural model greatly reduces ambiguity and improves confidence in the data interpretation, however this adds to the complexity of the fitting procedure. Isotopic substitution or labelling is a practice used commonly by our research group [21–23] and the neutron community in general [18,22–24].

### 2.2. Data analysis—volume fraction approach

The neutron reflectivity profiles were analysed using RasCal, a MATLAB-based application specifically developed for the analysis of neutron and X-ray reflectivity data (A. Hughes, ISIS Spallation Neutron Source, Rutherford Appleton Laboratory). RasCal employs an optical matrix formalism that allows users to fit an Abeles layer model to multiple datasets measured under different isotopic contrasts [17,19,25]. As indicated already, a successful fitting to multiple contrasts significantly reduces the possibility of ambiguity in data interpretation and provides reassurance in the model used.

For the Abeles layer method, an interface is described as a discrete series of homogeneous slabs characterized by their SLD and thickness [17,21]. The general solution to the optical matrix can also be modified to take into account interfacial roughness or density variations at each interface by implementing a Debye–Waller Gaussian-type roughness term, *σ*; derived by Névot & Croce [26,27]. Although the application of a Gaussian-type roughness factor does give a good approximation to the system investigated, this method of applying roughness does have notable drawbacks and may not be appropriate in cases where the density profile of a structure is not necessarily well represented by a Gaussian-like distribution.

In this study, an alternative to the Debye–Waller factor is derived, allowing for more elaborate structures or density profiles to be studied using a continuous density distribution model. The cuticular wax film distribution is represented, using a derived mathematical function. A convoluted Heaviside step function is used to model the volume fraction depth profiles for both the interfacial material and surrounding bulk at the solid–liquid interface. By determining the fractional contribution of the interfacial material and bulk water, a continuous profile of varying SLD was derived.

The constructed SLD profile is sliced into unroughened, infinitesimally small slabs of discrete SLD, before being stitched together and resampled using the standard Abeles layer method. This method produces a comprehensive reflectivity profile that can accurately represent the material's SLD versus interfacial distance relationship in terms of thickness, *τ* and a Heaviside decay (roughness) parameter, *σ*. The Heaviside function is described in electronic supplementary material, section A.

A least-squares minimization (Nelder–Mead simplex) was used to compare the reflectivity models with the experimental data and determine the best-fitting parameters; thickness, SLD and Heaviside decay parameter. In all cases, the simplest possible model that satisfactorily described the data was selected, and samples under different isotopic contrasts were constrained to be fitted to the same structure profile (layer thickness and decay parameter)—within error—with only the SLD from the different water contrasts varied to allow for the determination of hydration and surface coverage. The standard errors associated with each measurement were determined using a bootstrap resampling algorithm built into the RasCal fitting software. The fitted parameters and their associated errors were further verified by using an algorithm based on Bayesian probability theory that was found to be largely comparable to the treatment method used in this study, increasing confidence in the model used [28].

## 3. Experimental section

### 3.1. Wax extraction

Two reconstituted cuticular wax film samples were produced from waxes extracted from field-grown and laboratory-grown wheat straw, respectively. Cuticular waxes from harvested bulk agroresidue *Triticum aestivum* L. (wheat) straw were extracted on a large scale using supercritical CO_2_. These films will be denoted by the expression ‘SCW’.

Films formed from two-week-old wheat straw grown in controlled laboratory conditions were extracted via dissolution in chloroform and these reconstituted films will be denoted by ‘laboratory-grown wheat’ (LGW). Chloroform extraction is widely used to extract cuticular waxes. LGW waxes were used as a control to compare the influence of the growing conditions and extraction method on the reconstituted model wax films produced. Sabre wheat variety of wheat straw was used for both extraction techniques.

#### 3.1.1. Laboratory-grown wheat waxes—extracted by chloroform dissolution

Wheat samples were seeded in three separate plant pots and grown for 14 days at 20°C under fixed humidity conditions and in natural daylight. Waxes were extracted by dipping the leaf blades in chloroform for 30 min at room temperature (20°C) before the extract was filtered (Whatman filter paper, 150 mm in diameter, 11 µm in pore size) and solvent removed under vacuum until there were no detectable changes in weight, leaving behind only the extracted waxes. A dissolution time of 30 min is reported to recover approximately 90% of waxes present in the leaves of cereal crops (i.e. wheat, barley), with samples found to be largely free of phospholipids and chlorophyll, indicators of contamination from lipids within the leaf [7,28].

#### 3.1.2. Field-grown waxes—extracted by supercritical CO_2_

Supercritical CO_2_ extraction of wheat waxes was carried out from a pilot plant developed, and the full details are given by Deswarte *et al*. [10]. Along with the environmental and toxicological benefits, the main technical advantage of this method over more traditional solvent extraction techniques is the selective extraction on a large scale. The fraction of unwanted compounds can be minimized by tuning the reaction time, temperature and pressure and therefore the solvent strength. Thus, a maximum yield of cuticular waxes can be isolated predominantly without unwanted co-extractives such as polar lipids or sugars [10]. Furthermore, no solvent residue is left behind, and no further purification is required. The waxes used in this work were extracted by supercritical CO_2_ at pressure and temperature conditions of 100 bar and 40°C for 100 min, at a constant flow rate of 5 kg h^−1^ to give a wax yield greater than 0.4% w/w of the total wheat straw. Harvested field-grown wheat straw (Sabre variety) was milled into particulates of 0.5–5 mm before wax extraction.

### 3.2. Sample preparation and thin film coating

Model-reconstituted wax films were coated onto an optically flat silicon (Si) substrate to facilitate physical measurements. Prior to coating, the polished Si block surface underwent Piranha cleaning with a H_2_SO_4_ : H_2_O_2_ ratio of 3 : 1 at 90°C for 10 s, before being meticulously cleaned with 5% Decon-90 solution (Decon Laboratories Ltd) using lint-free tissue and rinsed with copious amounts of deionized water (Purelab UHQ, Vivendi Water Systems Ltd) and dried under nitrogen gas. After cleaning, the native oxide layer (SiO_2_) present on the Si surface was measured using ellipsometry. Using this cleaning method, the native SiO_2_ layer was found to have a reproducible thickness of 12–15 Å.

The wheat wax samples from both extraction methods were dissolved in chloroform (greater than 99%, Sigma Aldrich Co. Ltd) at a concentration of 0.05% w/w. Both solutions were placed in an ultrasonic bath for 15 min to assist dissolution. Reconstituted ultrathin wax films were then produced by depositing 1 ml of wax solution onto the Si block (50 mm× 60 mm× 1.2 mm), held in place with vacuum under nitrogen conditions before beginning the spin-coating procedure (Laurell Technologies, model WS-650MZ-23NPP); with the process programmed to run for 20 s at 3000 rpm including an initial acceleration of 1000 r.p.m. for 3 s. The above-mentioned method was found to generate reproducible uniformly coated films, both within the thickness range of cuticular wax films found in cereal crops and within the range ideal for NR measurements (discussed in electronic supplementary material, section B). Contact angle measurements supporting the study were also carried out by comparing the water contact angle upon reconstituted wax films formed from spin-coated SCW waxes with that of the adaxial (top) surface of an LGW leaf (electronic supplementary material, section C).

### 3.3. Neutron reflection

Specular NR measurements were carried out using the time-of-flight reflectometers D17 at ILL, Grenoble, France [29] and INTER at Rutherford Appleton Laboratories (RAL), Didcot, Oxfordshire, UK [30,31]. To create a sample environment for measurements at solid–liquid and solid–air interfaces, a Perspex trough with inlet tubes for liquid flow (1.5 ml volume) was clamped to an Si block of the same dimensions by means of a steel assembly to form a purpose-built sample cell. The sample cell was attached to a high-performance liquid chromatography pump (Hitachi/LaChrom, RS232 configuration) via inlet tubes to allow for regulated solvent exchange between measurements. This allowed wax samples under solid–liquid conditions to be measured under multiple water contrasts. To ensure full solvent exchange, 10 ml of solution was pumped through the trough at 2 ml min^−1^. Flow was controlled at this rate to ensure that the wax films were unaffected by the shear forces associated with the flow.

For moisture-controlled solid–air investigations, samples underwent additional preparation steps. For waxes measured in conditions mimicking a dehydrated environment, a freshly coated block was placed in a vacuum oven (Thermo Scientific, min. pressure 0.01 mbar) overnight at 25°C to remove moisture trapped within the film before being sealed in the liquid flow-cell assembly. For measurements in conditions mimicking a fully saturated environment, the Si-block-flow-cell assembly was connected via its inlet tubes to a sealed D_2_O reservoir at 40°C. The reservoir was in turn connected to a low-pressure nitrogen supply to encourage the evaporation of water vapour at elevated pressures from the reservoir and into the liquid cell. Saturation was determined to be at the point at which water droplets could be detected within the liquid cell.

On the INTER reflectometer, all measurements were carried out using a variable angle sample stage at 0.70° and 2.3° to the incoming beam, providing a wide Q-range of 0.008–0.35 Å^−1^ with a detector resolution of 3%. On the vertical reflectometer D17, measurements were carried out at 0.8° and 3.2°, with a Q-range of 0.008–0.16 Å^−1^ and 7% resolution. The same set-ups were used for the solid–air and solid–liquid measurements. All measurements were carried out at ambient temperature (298 K). While the majority of the investigation was carried out on INTER, the D17 instrument at ILL proved valuable owing to its use of a two-dimensional detector, which could help observe off-specular scattering signals occurring from roughened surfaces [32].

### 3.4. Spectroscopic ellipsometry

NR measurements were supported by SE measurements (J.A. Woollam Co., Inc.) in both dry and liquid conditions. SE measurements were carried out upon 2 cm× 2 cm spin-coated Si wafers prepared in a similar fashion to those used for NR. Under dry solid–air conditions, samples were measured at multiple angles (65°, 70° and 75°) over a wavelength range of 200–1000 nm. Data from all three angles were simultaneously fitted to minimize errors and increase the precision of results. Measurements were repeated on multiple films and in multiple positions on the substrate to confirm reproducibility. For solid–liquid measurements using a liquid-cell arrangement (500 µl Variable Temperature LiquidCell™, J. A. Woollam Co., Inc.), scans were carried out upon a wax-coated Si wafer at a fixed angle of 70°.

### 3.5. Atomic force microscopy

AFM measurements carried out upon reconstituted films formed from SCW and LGW waxes were undertaken at RAL, Harwell Campus. Scans were carried out in tapping mode using a gold-plated Si cantilever of tip height of 10–15 µm and tip curvature of radius 10 nm (NT-MDT, NSG10). Reconstituted wax samples for AFM were coated onto an Si substrate surface (6 cm× 5 cm) in the same way as for NR (3000 r.p.m., 20 s).

### 3.6. Scanning electron microscopy

Cryo-SEM images of model wax films formed from SCW extracted waxes were compared with images of the adaxial surface of an excised two-week-old laboratory-grown wheat leaf. A spin-coated Si wafer (50 µl, 0.05% wax-in-chloroform solution deposited) and excised leaf pieces (both *ca* 10 mm^2^) were mounted on 5 mm high, 10 mm diameter Jeol aluminium specimen stubs with sticky carbon tabs secured in a Quorum PP3010T specimen shuttle by means of a clamping screw. The specimen shuttle was located on a Quorum PP3010T gas-cooled stage in a Hitachi SU8220 field emission scanning electron microscope and maintained at a temperature of −60*°*C. Samples were observed at an accelerating voltage of 2 kV and working distance of approximately 7 mm (full information in electronic supplementary material, section D).

## 4. Results and discussion

### 4.1. Imaging analysis

**Figure 1.**
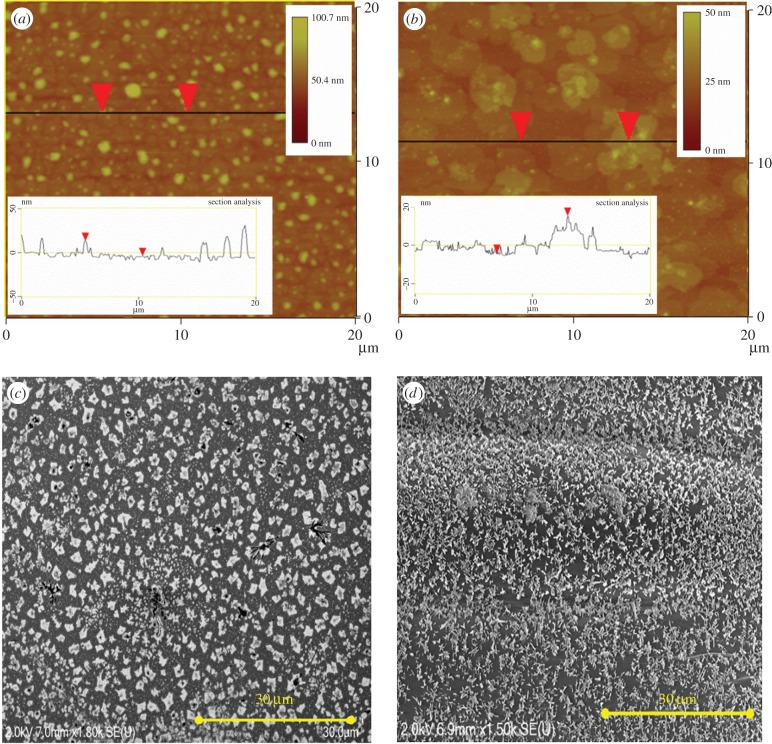
(*a,b*) 20 μm× 20 µm scans of wax films formed from field-grown waxes extracted by supercritical CO_2_ (SCW) and laboratory-grown waxes, extracted via dissolution in chloroform (LGW), respectively. Scan shows the two-layer extrusion-film structure. Mean roughnesses of scanned areas were found to be 7.7 and 4.6 nm for the SCW and LGW films, respectively. (*c,d*). Cryo-SEM images of a reconstituted wheat wax film surface (SCW) and that of an adaxial surface of an excised wheat leaf (two-week-old glasshouse-grown plant; scale bars, 30 µm).

AFM imaging was carried out upon model films formed from waxes of field-grown wheat straw extracted by supercritical CO_2_ (SCW) and also of wheat straw grown in controlled laboratory conditions extracted via dissolution in chloroform (LGW), with representative images shown in figure 1*a*,*b* over a 20 µm*^2^* region. In contrast, cryo-SEM imaging was carried out upon a reconstituted wax film formed from SCW extracted waxes and upon the adaxial surface of an excised two-week-old wheat leaf (figure 1*c*,*d*).

Both imaging techniques reveal a thin, uniformly coated underlying film with micro- and nanoscale extrusions present on the outer surface of each film. Cryo-SEM images of the surface of the supercritical CO_2_-extracted wax film and that of the adaxial surface of an excised wheat leaf appeared to be of a very similar structure and uniformity. On average, the surface extrusions distributed on the excised leaf's cuticular wax film appear denser, although both examples do show regions of varying density across the scanned surface. Overall, the model film prepared provides a physical model system that bears a good resemblance to the actual plant surface and provides a good starting point for further study.

In addition, AFM scans show that the reconstituted wax films obtained from the supercritical CO_2_ extraction display sharp, regularly distributed surface features reaching up to approximately 500 Å in height. In contrast, model films formed from extracted LGWs bear crystalline extrusions that appear larger and more diffuse. In spite of smaller heights (max height values approx. 350 Å), the micro- and nanoscale extrusions occupy a significantly larger surface area than that found on the reconstituted wax films formed from the supercritical CO_2_ extraction, but it remains unclear at this stage whether such difference is general and widespread.

The sharper ‘crystals' found on the surface of the SCW wax film in figure 1*a* have a mean radius of 290 nm with an average separation between extrusions of 2.9 µm. A roughness analysis over the measured wax surface gives the root-mean-squared (RMS) roughness of the surface extrusion layer to be 7.7 nm. As already indicated, the surface features from LGW extracted wax films are both larger and smoother with an approximate crystal radius of approximately 750 nm. The LGW film was therefore observed to have a denser, yet flatter extrusion layer with an RMS roughness of 4.6 nm. Despite this difference, the sectional analysis plots shown in figure 1*a,b* show that the wax volume fractions and distribution profiles within the extrusion layer of both films are comparable (electronic supplementary material, section E).

Many factors can affect the morphological appearance of a leaf surface and the same must be true for the reconstituted SCW and LGW wax films. Apart from the growing conditions, method of extraction and the type of solvents can also affect the chemical composition of the extracted waxes [10]. Use of chloroform and supercritical CO_2_ to extract waxes may well lead to some differences in the extracted wax composition which can also differ from those obtained from alternative techniques such as enzymatic wax extraction approaches [6,9–11,33]. Additionally, as described by Bianchi and Riederer, among others, even for the same species of crop, environmental factors, seeds and age affect the plant growing conditions [5,6,34,35]. In nature, drastic variations in height, colour and health can be observed from leaf to leaf, which could be attributed to a whole host of variables including the distribution of light and water across the leaf, quality of the seed and nutrients in the soil. The difference in extraction techniques and growing conditions means that there is no single system that can characterize all cereal waxes. While these differences make it difficult to replicate structural and morphological details, the model wax films as described above show a representative two-layer structure, implying that the general appearance and surface morphology of the wax crystals is reproducible, and is a key feature of wheat wax films [36,37].

### 4.2. Neutron reflection

Films formed from LGW and SCW waxes were characterized in air, under various moisture-controlled conditions and at the solid–liquid interface under three solvent contrasts; D_2_O, water CMSi and water contrast matched to air (CMA). The successful fitting of the reflectivity profiles measured at the solid–air and solid–liquid interface will allow for an unambiguous determination of the wax film structure. In this work, we used a two-layer Heaviside step function model to describe the density distribution of both the underlying wax film and surface extrusion layers. In the following, we will describe the main outcome obtained from each of the techniques deployed.

#### 4.2.1. Neutron reflection: solid–liquid measurements

**Figure 2.**
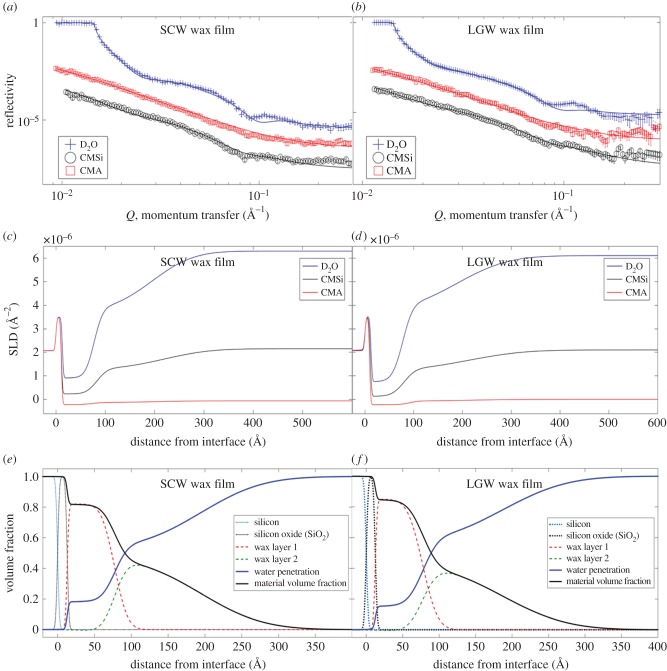
(*a,b*) Reflectivity plots for SCW and LGW wax films (normalized reflectivity versus momentum transfer, *Q*). Measurements were carried out under three solvent contrasts; D_2_O, water contrast matched to silicon (CMSi) and water contrast matched to air (CMA). For clarity, the CMSi and CMA reflectivity profiles have been scaled by ×0.01 and ×0.1, respectively. Error bars are smaller than symbols. (*c,d*) The associated SLD profiles describing the distribution of the wax films at the interface. (*e,f*) The volume fraction profiles of the wax films and water penetrating into the film as modelled by the Heaviside decay parameters. The corresponding best-fitted structural parameters to the reflectivity plots for SCW and LGW wax films are shown in tables 2 and 3, respectively.

The reflectivity profiles measured at the solid–liquid interface from reconstituted SCW and LGW wax films under three different solvent contrasts are shown in figure 2*a,b*, with their corresponding SLD and volume fraction profiles against interfacial distance shown in figure 2*c–f.* The wax films were modelled into the two layers representing the underlying film and outer surface wax extrusions, consistent with the main film structure observed from AFM and cryo-SEM. Within the constraints of the three isotopic water contrasts measured, as described in §2.1, the reflectivity profiles were fitted to a single best-fitted representative wax film model. The SLD profiles for both SCW and LGW wax films (figure 2*c,d* and tables 2 and 3) were calculated directly from their volume fraction distributions as discussed in §2.2. The results indicated that a significant amount of water penetrated into the underlying film layer; either through cracks or voids within the reconstituted film, or via a wax diffusion process. It was found that water makes up 18% and 15% of the total material at the silicon oxide–wax interface in the underlying wax layer of the two reconstituted SCW and LGW wax films, respectively. The volume fraction-distance profiles from both types of wax films show that the water content in the film increases rapidly from approximately 20% to over 50% across the upper 25–30 Å of the underlying layer (figure 2*e,f*), with the film's density profile modelled using the Heaviside decay parameter. Results point towards a highly diffusive and porous film susceptible to the transport of water and nutrients (figure 4*a*).

**Table 2.** Structural parameters obtained from solid–liquid NR measurements of model films produced from waxes extracted from field-grown wheat straw by supercritical CO_2_ (SCW). Measurements were carried out under three solvent contrasts; D_2_O, water contrast matched to silicon (CMSi) and water contrast matched to air (CMA). A two-layer Heaviside step function model (described in §2.2) is used to model NR profiles describing the underlying wax film and surface extrusion layers. The corresponding NR profiles are shown in figure 2*a*.

contrast	wax SLD at substrate (×10^−6^ Å^−2^)	wax SLD at film surface (×10^−6^ Å^−2^)	film layer		extrusion layer		combined reduced chi-sq.
			thickness, *τ*, (Å)	Heaviside decay parameter, *σ*, (Å)	thickness, *τ*, (Å)	Heaviside decay parameter, *σ*, (Å)	
D_2_O	0.90 ± 0.10	3.49 ± 0.10
CMA	−0.22 ± 0.05	−0.12 ± 0.05	65 ± 1	16.5 ± 1.5	97 ± 9	88.5 ± 5.0	25.8
CMSi	0.20 ± 0.10	1.06 ± 0.10

**Table 3.** Structural parameters obtained from solid–liquid NR measurements of model films produced from waxes extracted from laboratory-grown wheat straw by chloroform (LGW). Measurements were carried out under three solvent contrasts; D_2_O, water contrast matched to silicon (CMSi) and water contrast matched to air (CMA). A two-layer Heaviside step function model (described in §2.2) is used to model NR profiles describing the underlying wax film and surface extrusion layers. The corresponding NR profiles are shown in figure 2*b*.

contrast	wax SLD at substrate (×10^−6^ Å^−2^)	wax SLD at film surface (×10^−6^ Å^−2^)	film layer		extrusion layer		combined reduced chi-sq.
			thickness, *τ*, (Å)	Heaviside decay parameter, *σ*, (Å)	thickness, *τ*, (Å)	Heaviside decay parameter, *σ*, (Å)	
D_2_O	0.70 ± 0.15	3.43 ± 0.10					
CMA	−0.22 ± 0.10	−0.13 ± 0.05	71 ± 4	12.5 ± 2.0	105 ± 8	86.0 ± 4.0	13.0
CMSi	0.12 ± 0.10	1.03 ± 0.10					

The modelled wax reflectivity profiles were also found to correspond to the shapes expected if large micro- and nanoscale structures that were diffusely scattered upon the surface of the underlying film existed. This model is supported by the observation of the crystalline extrusions, using the various imaging techniques described. For both waxes, the wax volume fraction of the outer layer was modelled to gradually decrease from 50% at the outer surface of the underlying film to a negligible level at a distance of approximately 300 Å away from the silicon surface, with the shape of the wax volume fraction distributions mimicking that of the diffusely scattered surface features observed (figure 2*e,f*).

Both LGW and SCW wax films show very similar SLD and volume fraction profiles, again consistent with similar film structures obtained from AFM and cryo-SEM. Both wax films carry regularly distributed micro- and nanoscale extrusions that cause the off-specular or diffuse scattering of neutrons. As a qualitative indication of a diffusely roughened surface, off-specular scattering can be directly detected using a ^3^He tube multidetector on the D17 instrument at ILL. The two-dimensional detector profile reveals a single strong reflected diffraction peak referred to as a ‘Yoneda wing’ (electronic supplementary material, section F). Yoneda scattering is a result of microscale roughening of an interface leading to diffuse scattering of neutrons near the critical angle of total reflection [17,32,38,39]. The critical angle limit, *Q*_c_, is defined by the following equation4.1
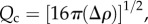
where Δ*ρ* = *ρ*_1_ – *ρ*_2_; *ρ*_1_ and *ρ*_2_ are the SLDs for the two bulk phases. For a silicon–D_2_O interface (*ρ*_1_ – *ρ*_2__=_ *ρ*_D_2___O_ – *ρ*_silicon_), the critical angle limit of total reflection is seen at 0.0147 Å^−1^ (figure 2*a,b*). Under CMA or CMSi conditions, a critical edge is not observed as *ρ*_1_ is smaller than or equal to *ρ*_2_, causing the critical angle to be imaginary.

Assuming a smooth, flat interfacial layer, the reflectivity profile at the silicon–wax–D_2_O interface would undergo total reflection, with reflectivity normalized to 1 below the critical angle limit. While not clearly seen in figure 2*a,b*, the critical edges of the two profiles are both smeared and reduced, leading to reduced specular reflectivity values for the three measured contrasts (the actual reflectivity profile for the SCW wax film is scaled to 0.81 ± 0.03 by taking these effects into account). The results have revealed that for SCW wax films 19% of the incoming beam was lost to the off-specular scattering arising from the ordered micro- and nanoscale extrusions present upon the film surface.

Conversely, the crystalline-like extrusions found upon the surface of the LGW wax films are considerably less sharp and ordered than the corresponding SCW films leading to a smaller off-specular reflection contribution. The reflectivity values around the critical edges measured from LGW wax films (normalized to the incident flux) were found to be 0.93 ± 0.05. Despite the difference, the presence of the crystalline-like wax extrusions did not appear to influence water penetration through both films.

#### 4.2.2. Neutron reflection: solid–air measurements

**Figure 3.**
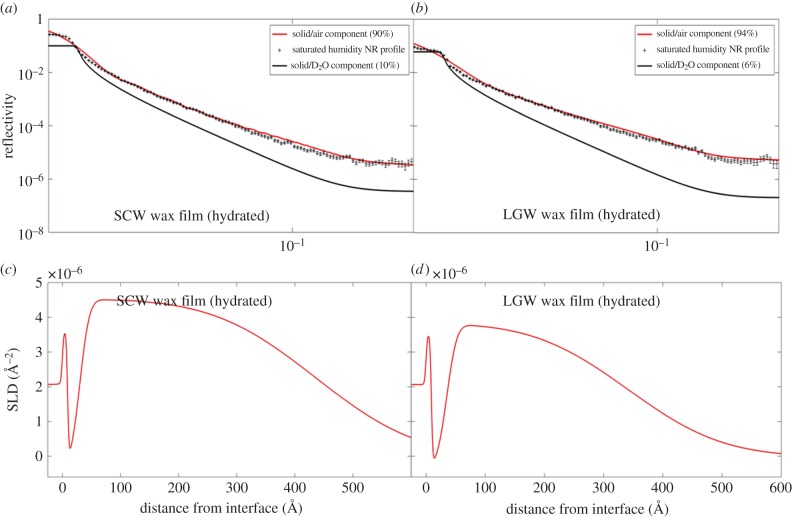
(*a,b*) The contributing solid–air and solid–D_2_O reflectivity components that are incoherently summed to obtain the final reflectivity profiles for the SCW and LGW wax films measured under saturated humidity conditions. A two-layer Heaviside step function model (described in §2.2) is used to model NR profiles describing the underlying wax film and surface extrusion layers. (*c,d*) The associated SLD profiles. The corresponding best-fitted structural parameters are shown in table 4.

Solid–air NR measurements carried out under various humidity conditions (figure 3 and electronic supplementary material, figure S5), provided additional structural information to complement the solid–liquid study as described above. Owing to the lack of scattering contrast between the SLD of air and that of the waxes, limited information could be obtained using neutron reflectivity under dry conditions. However, as the scattering contrast of D_2_O is significantly different to that of the waxes and air, introducing moisture in the form of D_2_O to the wax films would allow for useful assessment of the effect of hydration upon the wax films.

**Table 4.** Structural parameters obtained from solid–air NR measurements of SCW and LGW model films under fully hydrated moisture conditions. The corresponding NR profiles are shown in figure 3*a,b* respectively. A two-layer Heaviside step function model (described in §2.2) is used to model NR profiles describing the underlying wax film and surface extrusion layer.

sample	wax SLD at substrate (×10^−6^ Å^−2^)	wax SLD at Film surface (×10^−6^ Å^−2^)	film layer		extrusion layer		chi-sq.
			thickness, *τ*, (Å)	Heaviside decay parameter, *σ*, (Å)	thickness, *τ*, (Å)	Heaviside decay parameter, *σ*, (Å)	
SCW-hydrated	−0.3 ± 0.20	4.23 ± 0.20	20 ± 4	14 ± 3.0	393 ± 30	152 ± 15	8.91
LGW-hydrated	−0.3 ± 0.20	3.56 ± 0.20	27 ± 2	17 ± 2.0	308 ± 20	126 ± 5	8.67

**Figure 4.**
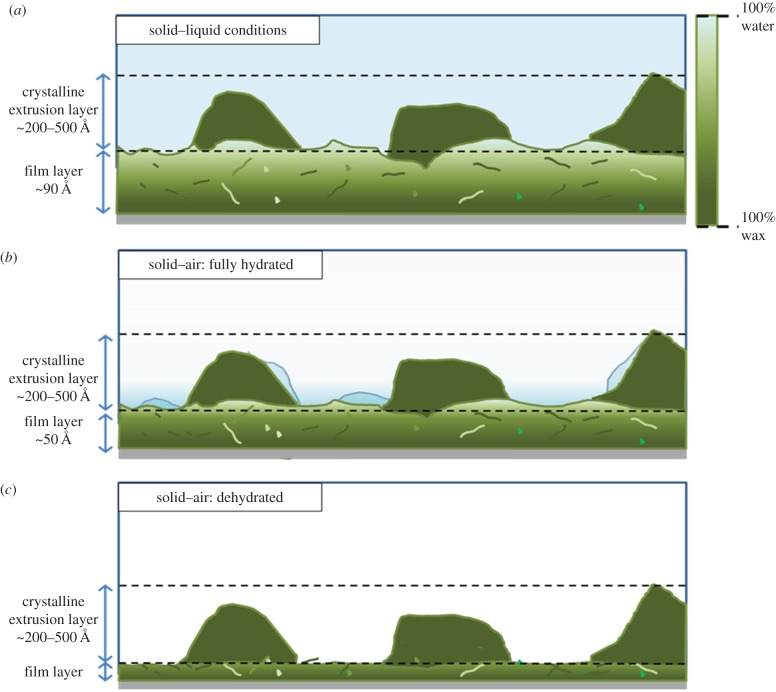
(*a–c*) Representative cartoon depicting a tomographic image of a wax film under various conditions. (*a*) A swollen wax film immersed in water caused by water penetration. The colour gradient describes the amount of water penetrating the wax film. (*b*) The wax film in a moisture-saturated environment. The underlying film undergoes swelling; however, the amount of water penetration of the film is significantly smaller. Condensed water and water vapour are also present on the film surface. (*c*) A shrivelled wax film in dehydrated air conditions. It is assumed that the basic structural feature of the wax crystalline extrusions is unaffected by the various conditions.

Measurements carried out in a moisture-saturated environment showed that it is particularly easy for water vapour to penetrate and become trapped within the underlying wax films. The moisture-saturated profiles for the SCW and LGW wax films both show a steady increase in water content with interfacial distance as the water vapour penetrates the wax more readily near the surface of the underlying film (figures 3*c,d* and 4*b*), thus causing the film to swell. For SCW waxes, a thickness of 20 Å and a significant decay parameter of 14 Å were found to accurately model the underlying film structure. The distribution of water present within the porous film, mathematically described by the Heaviside decay parameter, showed that water penetration reduced the wax coverage near the film surface to 32% of the total film. For LGW, the wax coverage at the top of the underlying film layer of 27 Å in thickness and with a decay parameter of 17 Å was 42% (table 4). Similar to what was seen for the solid–liquid profiles, albeit to a lesser extent, this result demonstrates that water molecules can easily swell and penetrate the wax film layers.

The SLD profiles (figure 3*c,d*) also show that some condensed water is present within the defined surface extrusion layer. The volume fraction of water within this layer is observed to decrease with interfacial distance, in line with what would be expected from patches of condensed water upon the film surface. This observation resulted from the sample control as the moisture-saturated water environment was considered to be reached at the point where condensation was visually observed within the liquid cell. The formation of some visible droplets upon the substrate surface explain the complex SLD distribution as shown in figure 3, where a gradual reduction in the average water content from approximately 35% of the total volume fraction at the underlying film surface to the negligible level at the top of the extrusion layer can be seen. The effects of the condensed water and vapour upon the wax film surface are illustrated in figure 4*b*.

The presence of the condensed D_2_O upon the film surface also explains the ‘smeared’ low reflectivity critical edges observed for the moisture-saturated samples (figure 3*a,b*). The condensed water droplets are of a large enough length scale (greater than the neutron coherence length, 5–50 µm) to act as a bulk phase to the scattering neutrons upon the film surface, resulting in the reduced critical edge. Furthermore, the smeared critical edges are indicative of crystalline structures present on the film surface on micro- and nano length scales. In order to fit the reflectivity profile, the incoherent sum of the two reflectivities coming from the condensed water, and water-free regions of the wax surface would have to be considered (electronic supplementary material, section H). By carrying out a scaled sum of the reflectivities, the condensed water fraction upon the film surface was found to be 10% for SCW and 6% for LGW wax films. When comparing the contributing reflectivity components with their incoherent sum (figure 3 and electronic supplementary material, figure S6), it is clear that the incoherent contribution to the reflectivity from the condensed water has very little influence upon the final model.

Despite moisture penetration and subsequent swelling, the hydrated wax films are significantly thinner than that immersed in water at the solid–liquid interface. From the SLD plots against the interfacial distance at solid–air and solid–liquid interfaces, it can be seen that exposure to the bulk water caused more substantial swelling to the underlying wax film, evident from the dramatic increase in the film thickness, *τ* and smearing of the film-extrusion boundary (represented by the roughness parameter, *σ*; figures 2 and 4).

Measurements at the solid–air interface were also carried out in both ambient and dehydrated environments (electronic supplementary material, section G). Results show that wax profiles measured under dehydrated and ambient conditions can be fitted to the same two-layer model, suggesting that dehydrating the wax film had no notable effect upon the basic wax film structure feature. As the SLD contrasts between the waxes and air are poor, any fitting models applied suffer from the large errors associated. Based on our results shown above, it can however be inferred that a dehydrated reconstituted wax film will be significantly thinner than its hydrated counterpart. To our best knowledge, this is the first study to report the swelling and dehydrating of the cuticular plant wax films as a way to control water transport from the reconstituted wax films. It is highly likely that the same mechanism works across plant surfaces.

### 4.3. Spectroscopic ellipsometry

Using SE, the wax-coated films could be modelled to describe the refractive index–wavelength relationship in both solid–air and solid–liquid conditions. Wax films were initially fitted as a uniform layer with a fixed refractive index of *n* = 1.47 (refractive index common for fatty, wax-like substances) with an associated exponentially decaying extinction coefficient, *k* in the UV–visible region which becomes negligible greater than 400 nm (electronic supplementary material, section I). This model yielded a wax film thickness of 75.0 ± 5.0 Å, with a mean-squared-error (MSE) value of 4.48 to describe the goodness of the fit [40]. Unlike NR, however, SE, while useful to obtain a coarse thickness estimate, suffers from the coupling of refractive index and thickness parameters over the range studied and thus cannot be used to accurately decouple the underlying film thickness and composition. As a result, the film was modelled as a single homogeneous equivalent layer, interpreting the wax film as a single slab. As a confirmation of the uniformity and approximate size of the underlying thin film coating, however, SE plays a very useful role.

For SE measurements at the solid–liquid interface, the thickness of the film layer was determined to be 120 Å (MSE = 6.63), significantly thicker than that measured in air using the same uniform layer model. This suggests that the total film thickness appears to increase under liquid conditions owing to water penetration and swelling. To further investigate this observation, a more representative linearly graded model was created to model the effects of water penetration. A best-fitting model proposing up to 65% water penetration at the wax surface, decreasing linearly to 0% at the wax–silicon interface was found to give an MSE of 5.90 for a single homogeneous film layer of 156 Å. The thickness of a fully immersed wax film was found to roughly double, in agreement with NR.

## 5. Conclusion

The epicuticular wax film structure of *T. aestivum* L. (wheat) consists of an ultrathin, underlying film with characteristic micro- and nanoscale extrusions on the outer surface. Using NR, AFM and cryo-SEM and confirmed via ellipsometry, the two reconstituted wax films were found to consist of the same structural features, comprising an underlying wax film and outer crystalline extrusions. This work has thus demonstrated that the main leaf surface could be mimicked in its main characteristics by reconstituted films, thus opening up the prospect for further investigations using cuticular wax models by elaborate physical techniques.

The general perception is that plant cuticular waxes form part of the hydrophobic, waterproof surface coating whose primary function is to act as a water-repellent barrier against external environmental attacks. While this is apparent on the macroscopic scale, our findings from the behaviour of reconstituted cuticular wax films show a significantly different behaviour at the nano- and molecular levels. While all combined studies support the formation of a thin underlying film containing micro- and nanoscale crystalline wax extrusions scattered upon the film surface, measurements carried out in moisture-saturated solid–air and solid–liquid environments point towards a highly porous and diffusive underlying wax film which allows a significant amount of water penetration, with up to 50% of the underlying film occupied by water. As a result, a strong swelling of the underlying wax film from approximately 50–100 Å was observed. These swollen wax films could return to their basic structural feature upon drying.

Cuticular plant waxes are known to comprise very complex compounds containing a small fraction of polar groups as well as various alcohols and hydrophobic components. It is possible that the composition of the wax films affects the wax structure in such a way that water and nutrients are given a pathway through the wax film over different size scales. This exploratory work of the epicuticular wax films could act as a springboard for further study into the role of the wax cuticular films in moderating the transport, uptake and losses of nutrients and water or agrochemicals through the plants. The influence of surfactants, known to significantly impact the penetration and transport of water and nutrients through leaf surfaces, can also be carefully examined to expand our current understanding of the mechanistic process across the wax films. A more reliable insight into the interfacial processes at the molecular level will help us to grasp the key factors affecting the actions of agrosprays and develop more efficient and effective pesticide formulations, crucial in satisfying the need for increasing food production and yield whilst reducing environmental impact.

## Supplementary Material

Supporting Information

## Data accessibility

Supporting NR data are available on request from the corresponding author. RasCal analysis software is available to download from https://sourceforge.net/projects/rscl/.
