# Supplementary material for: Structural features of reconstituted wheat wax films
Source: J R Soc Interface. 2016 Jul;13(120):20160396. doi: 10.1098/rsif.2016.0396 (PMC4971226; doi:10.1098/rsif.2016.0396)
Supplement: Supporting Information [file rsif20160396supp1.doc]

Supporting Information – Structural Features of Reconstituted Wheat Wax Films

*E. Pambou^a^, Z. Li^a^, M. Campana^b^, A. Hughes^b^, L. Clifton^b^, P. Gutfreund^c^, J. Foundling^d^, G. Bell^d^, J. R. Lu^a*^*

^a^ Biological Physics Group, School of Physics and Astronomy, University of Manchester, Oxford Road, Manchester, M13 9PL, UK.

^b^ STFC ISIS Facility, Rutherford Appleton Laboratory, Didcot OX11 0QX, U.K.

^c^ Institut Laue-Langevin, 71 avenue des Martyrs, 38000 Grenoble, France.

^d^ Syngenta, Jealott’s Hill International Research Centre, Bracknell, Berkshire, RG42 6EY, UK.

1. **Sample Preparation and Thin Film Coating**

The convoluted Heaviside step function developed to model the relationship between the wax film and surrounding bulk’s volume fractions with interfacial distance is described below:


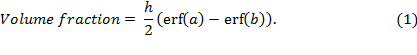


Where *h* is the height of the step function (normalized to ‘1’) and *erf(X)* is the ‘Gauss’ error function defined below. *a* and *b* describes the density distribution of a modelled layer. Here *x* is the distance from the interface while *τ_a_* and *τ_b_* describe the interfacial distances at the bottom and top of the reflectivity layer respectively.
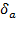
 and
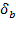
 are the fitted decay parameters at the start and end of the layer which define the shape of the error function.


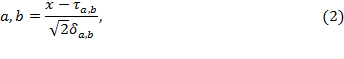


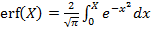
. (3)

The SLD profile constructed from the material’s volume fraction distribution can be sliced into unroughened, infinitesimally small slabs of discrete scattering length density and thickness, before being stitched together and resampled using the standard Abeles layer method to give a model reflectivity profile which can accurately describe the material’s SLD vs interfacial distance relationship. Under this approach, a more comprehensive model fitting requiring few variable parameters can be obtained to describe the film structure, compared to the standard roughness approach commonly used.

1. **Sample Preparation and Thin Film Coating**


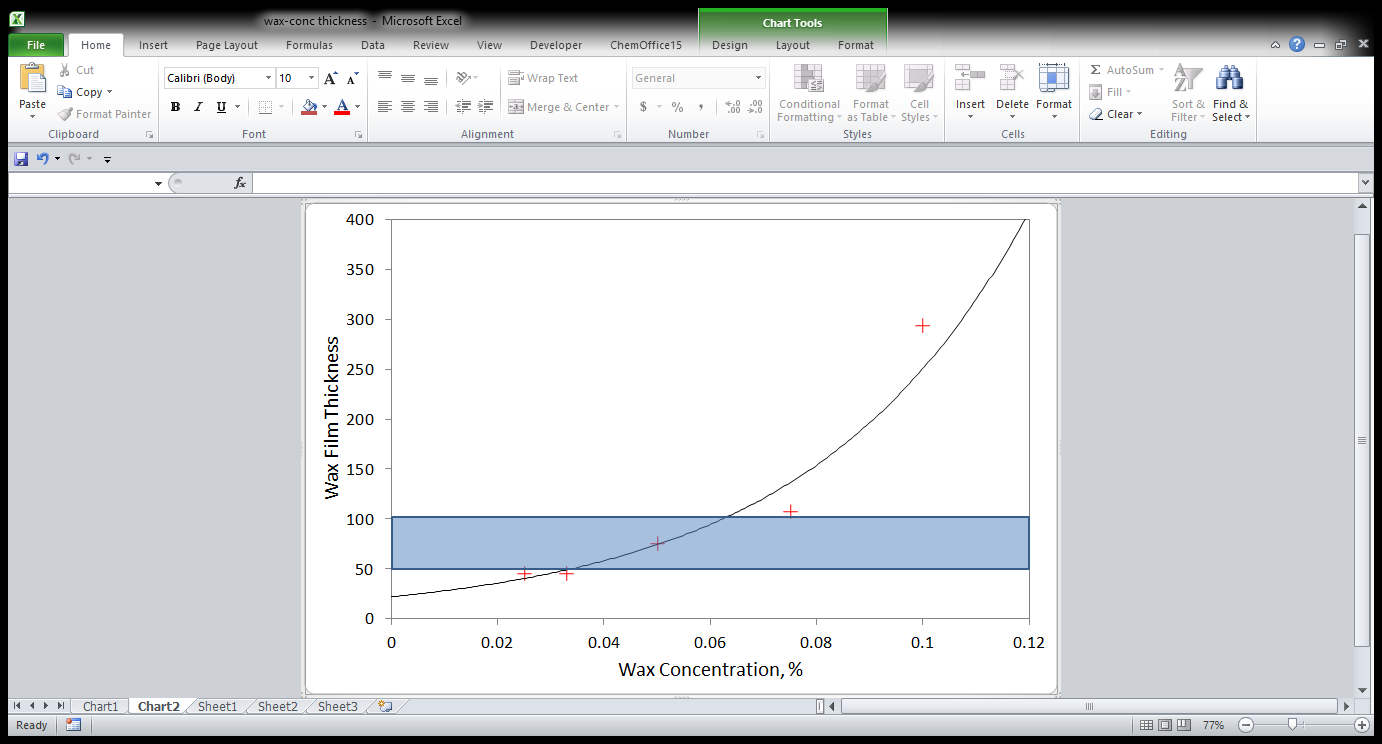


Upper limit- Precision of neutron reflection measurements decrease with film thickness

Lower limit of thickness for cuticular wax films.

Figure S1. Plot shows the relationship between concentrations of the wheat wax solution in chloroform (0.05% w/w) deposited onto a Si substrate vs. the thickness of the wax film produced

, Å

Wax films were fitted via ellipsometry using a uniform layer with a fixed refractive index of
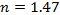
 (a predicted refractive index common for fatty, wax-like substances) with an associated exponentially decaying extinction coefficient, *k* in the UV-visible region which becomes negligible above 400 *nm.* The wax film is modelled as a single homogeneous equivalent layer. A concentration of 0.05 % w/w was sufficient to produce a reconstituted wheat wax film model.

1. **Contact Angle Measurements**

5 *μl* droplets of de-ionised water were deposited upon various surfaces, including a wax coated silicon block (60 *mm* × 50 *mm*), bare silicon, C8 (Octyltrimethoxysilane) treated surfaces for increased hydrophobicity (1), and the adaxial surface of a 3 week of wheat leaf. Table S1 shows the results of selected water contact angle measurements.

Contact angle measurements in-plane to the measured surface were carried out using a tripod mounted Nikon D3300 D-SLR camera with a Nikon AF-S Micro NIKKOR 60mm f/2.8G ED Lens attached. The ‘manual’ focus setting was used to ensure focus onto the deposited water droplet Samples were attached to a vibration table using double-sided tape to ensure maximum precision. The resultant PNG format files were analysed using the ‘angle’ tool of the free-to-use image processing and analysis software *ImageJ* to determine the water contact angle. Each sample was measured in 3 locations for increased accuracy. Using this set-up and methodology, errors of <± 2^o^ were observed.

| **Surface** | **Contact Angle, ±2 (^o^)** |
| --- | --- |
| Bare-clean Si | 10 |
| Wheat Wax coated Si | 61 |
| C8 Block treated Si | ^*^67 (± 4^o^) |
| Wheat Wax C8 coated Si | ^*^95 (± 4^o^) |
| Adaxial Wheat Leaf Surface | 150 |
| Annealed Si Block (Wheat coated) 1hr | 80 |
| Annealed Si Block (Wheat coated) 4hr | 88 |

Table S1: Table shows the selected results of contact angles.

Measurements show the excised adaxial wheat leaf surface to have a significantly higher contact angle than that of a reconstituted model wax film, with an observed trend relating the size of the contact angle with the hydrophobicity of the underlying substrate. Reconstituted wax films coated upon a hydrophilicSi-SiO_2_ interface were found to give a lower contact angle in comparison to a C8 treated Si surface or the excised wheat leaf surface.

It should be emphasised that a simple comparison of the water contact angle between excised leaf wax film samples and reconstituted wax films is not a reliable indicator of how representative the reconstituted model is. As with all cereal crops, the wheat leaf surface contains a variety of additional surface features not modelled by the reconstituted wax film which significantly affect the surface contact angles measured. The confocal microscopy image shows that on the micron scale; ordered ridges formed by the epidermal tissue, epidermal hairs and stomata are all distributed over the adaxial surface of the leaf; influencing the measured water contact angle. This has been supported by a number of high impact articles that report on how introducing intricately dispersed structures such as artificial ridges or needles to various metals fabrics and ceramics could significantly increase a materials hydrophobicity (2-4).


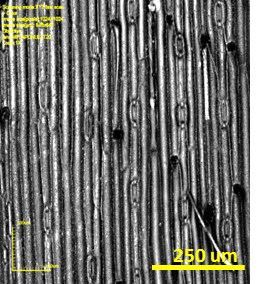


**Confocal Microscopy image of excised wheat leaf**

AFM and SEM scans carried out upon both excised and reconstituted wax films showed very similar film-extrusion structures which suggest that the underlying substrate hydrophobicity has little influence upon the ordering and packing of long chain molecules that form the wax film. Contact angle results do however support the paper findings in suggesting that water is able to penetrate the porous wax film. The differences in the water contact angles measured upon different substrate surfaces can be explained by the porosity of the reconstituted wax films which allow water droplets penetrate the film layer and come into contact with the underlying substrate, thus influencing the measured contact angle. This observation was further supported by the contact angle measurements carried out upon a reconstituted wheat wax film annealed for one and four hours respectively. The annealing process, carried out in order to strengthen the microstructure of the film and remove the pores within the film would be expected to reduce the amount of water penetration through the wax film as a result of this, thus increasing the water contact angle. This was observed.

1. **SEM Sample Preparation – Sample holder arrangement**

Excised leaf pieces (ca 10 *mm^2^*) were mounted on 5 *mm* high, 10 *mm* diameter *Jeol* aluminium specimen stubs with sticky carbon tabs. The stubs were secured in a *Quorum PP3010T* specimen shuttle, illustrated below, by means of a clamping screw (Fig. S2) –The specimen shuttle was located on a gas-cooled stage in a *Hitachi SU8220* field emission scanning electron microscope and maintained at a temperature of -60 *°C*.

Samples were observed at an accelerating voltage of 2 *kV* and working distance of approximately 7 *mm* using the upper secondary electron detector.

Specimen mount

**Quorum PP3010T Specimen Shuttle**

Sticky carbon tab

Leaf

Clamping screw


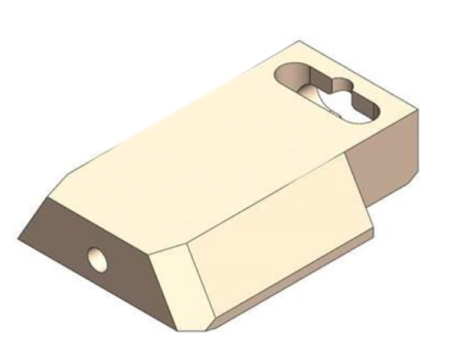

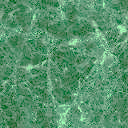


Figure S2. Schematic of Cyro-SEM setup

1. AFM: Depth analysis profiles


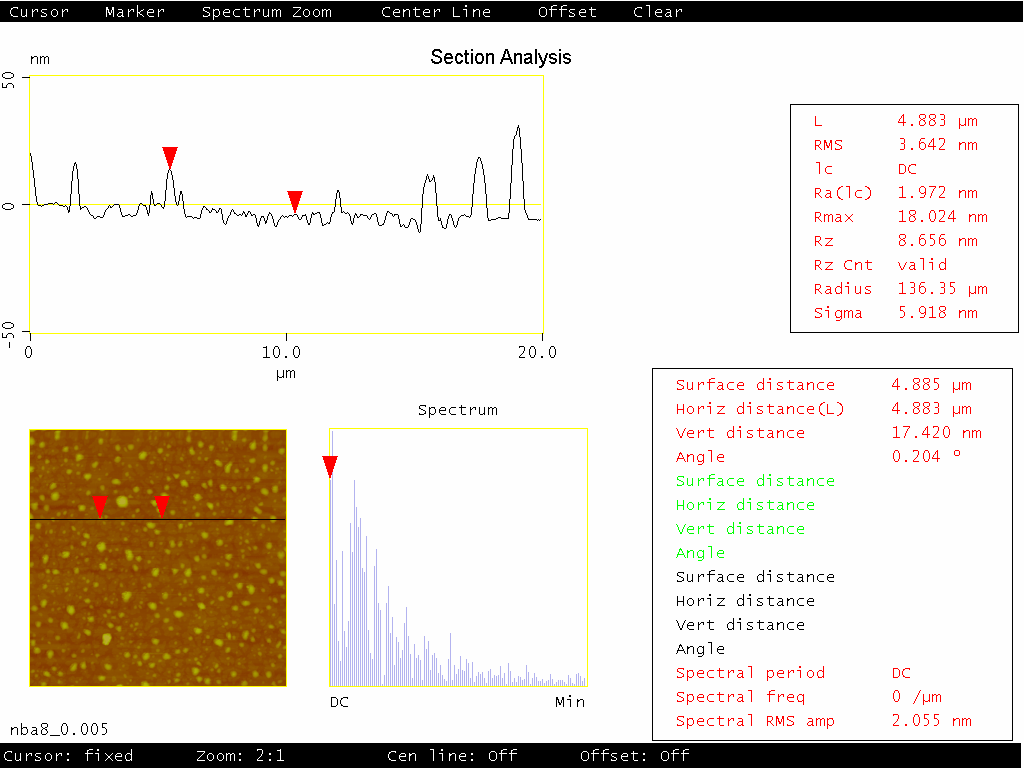

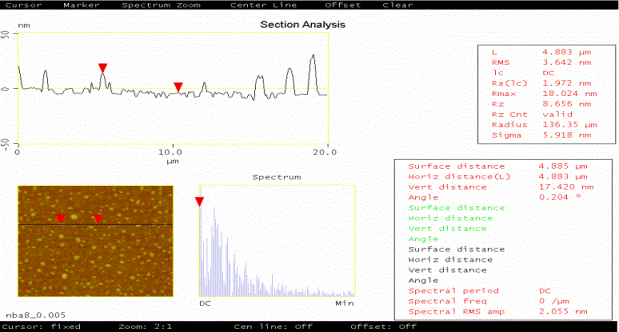

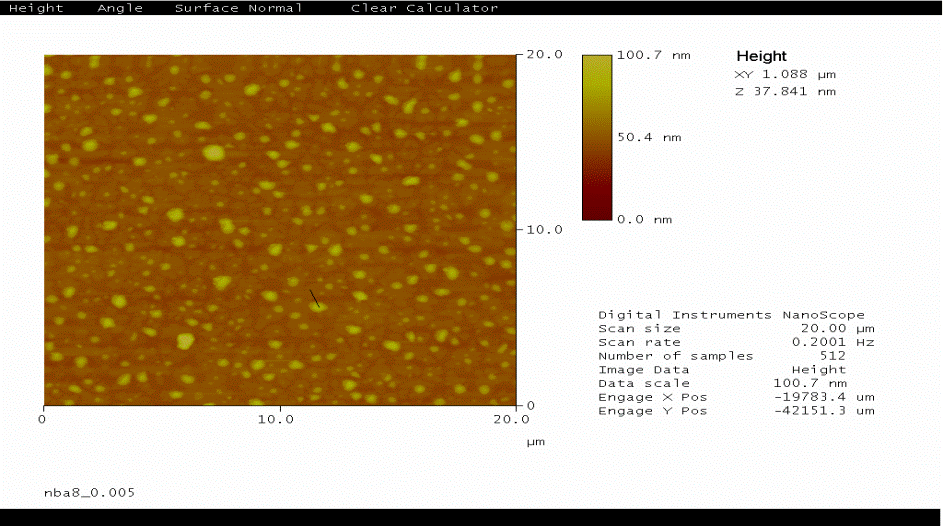

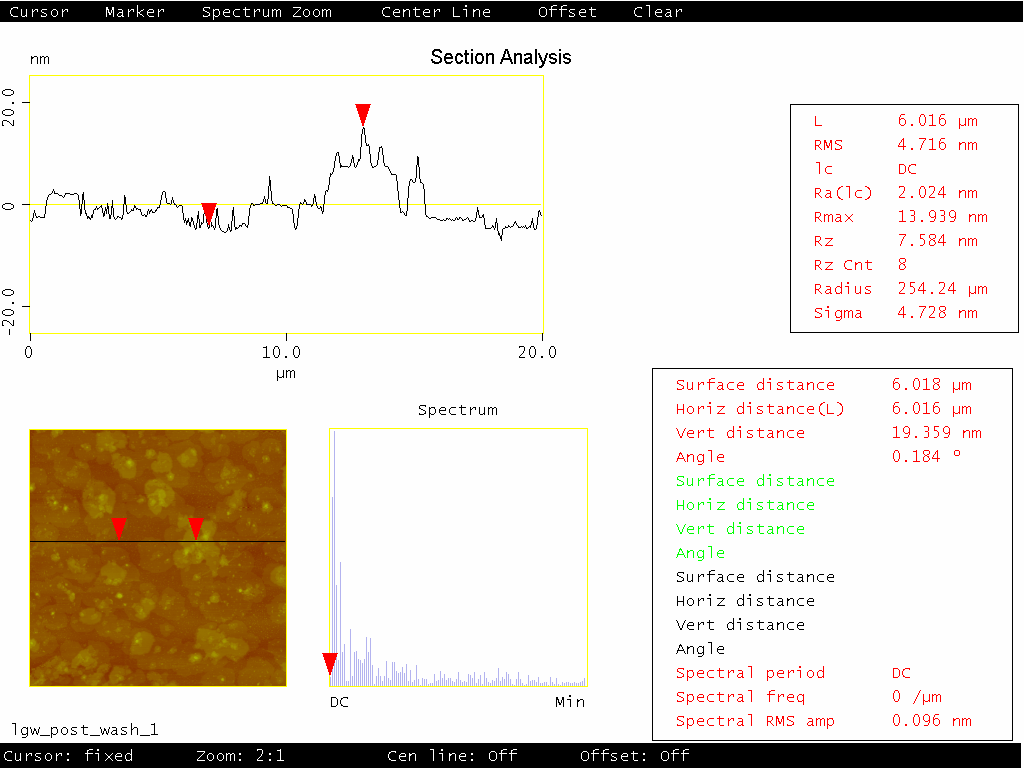

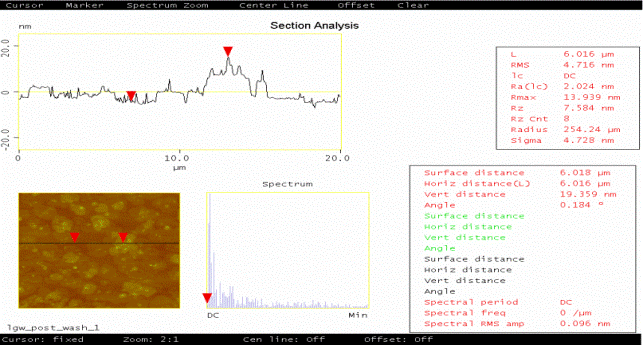

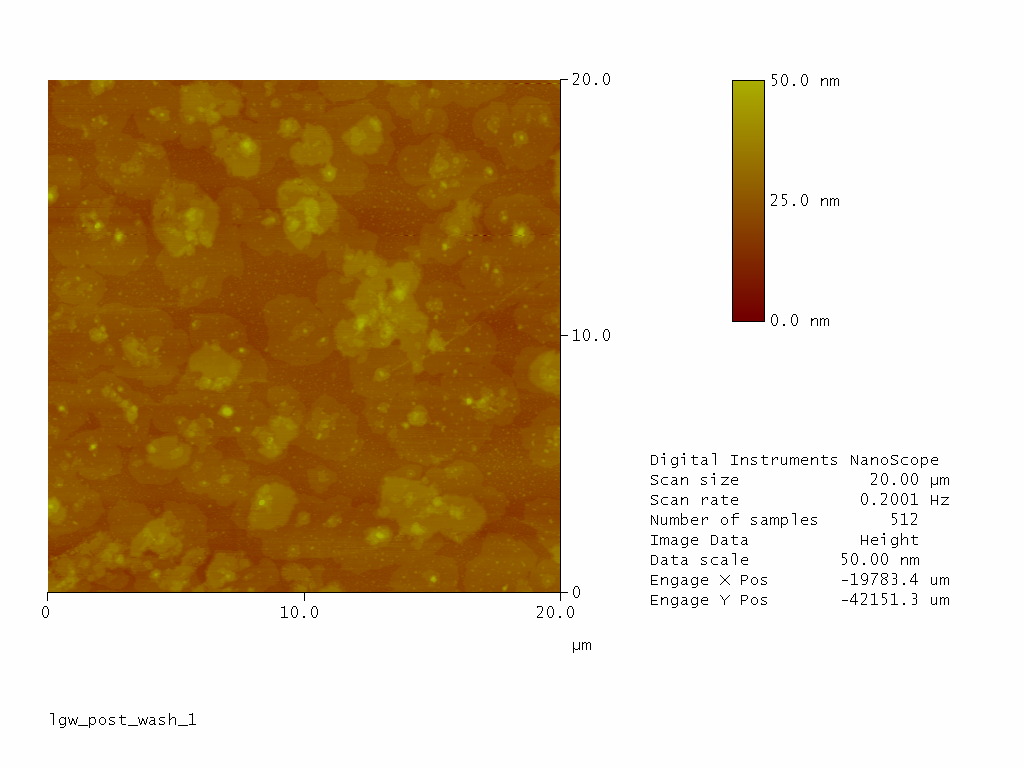

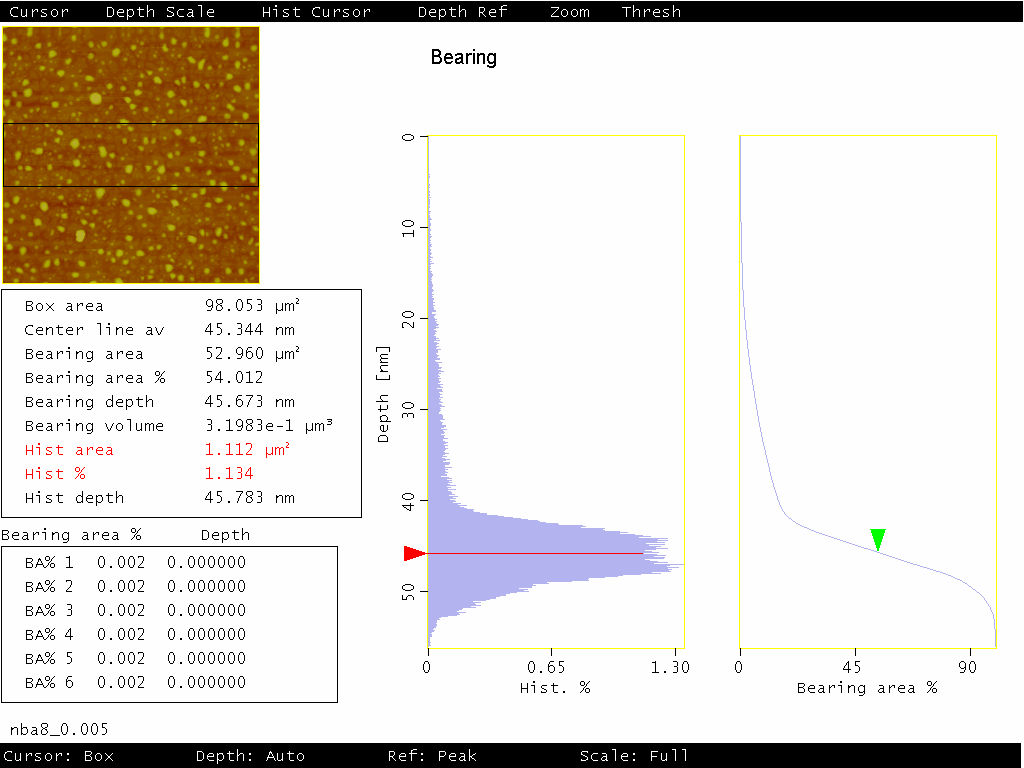

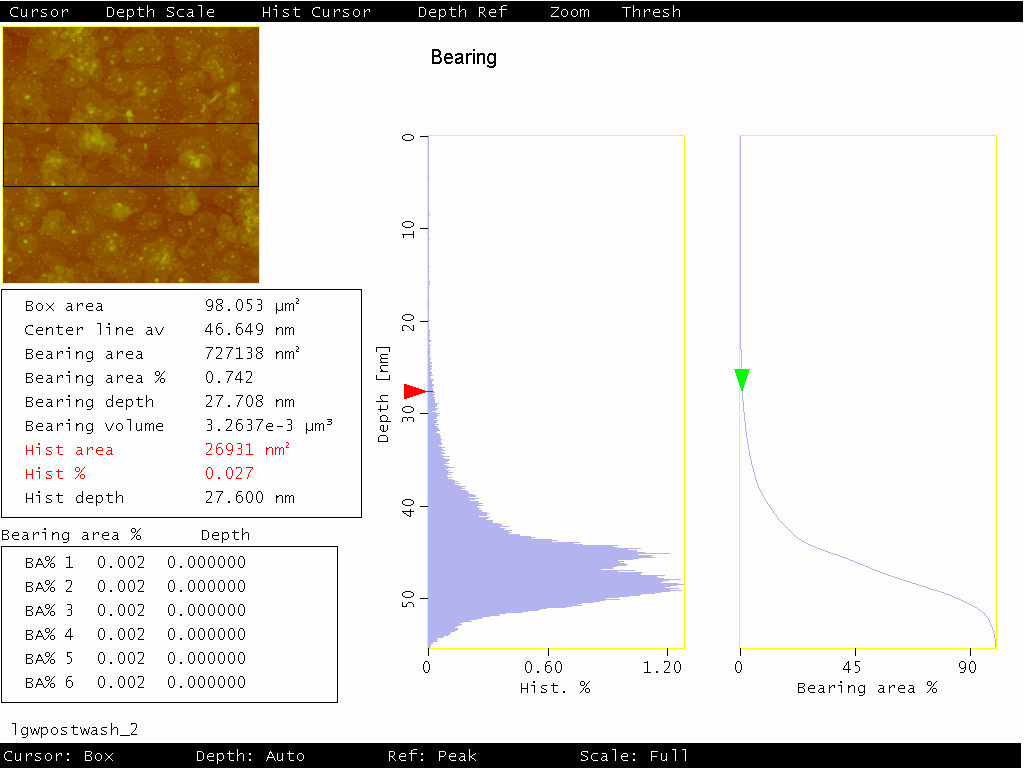


a.)

b.)

c.)

d.)

Figure S3. Figures S3c, d. profile the height variations across the 1D scan lines for the reconstituted SCW and LGW wax films respectively (Shown in Figures S3a, b). While the extrusions of the super-critically CO_2_ extracted waxes appear to be much sharper than the corresponding LGW extracted waxes - as described in the main text - the wax-height distribution profiles are approximately similar for both waxes; with both waxes showing an average film height of ~12 *nm*. This is comparable to the one layer thickness model as determined by ellipsometry.

**Fig. S2)** a.) and b.) are the AFM images for SCW and LGW wax samples (. Fig. c.) and d.) are their associated depth distribution profiles, describing the variation in surface height of the waxes.

1. Yoneda Wings: D17 2D Detector


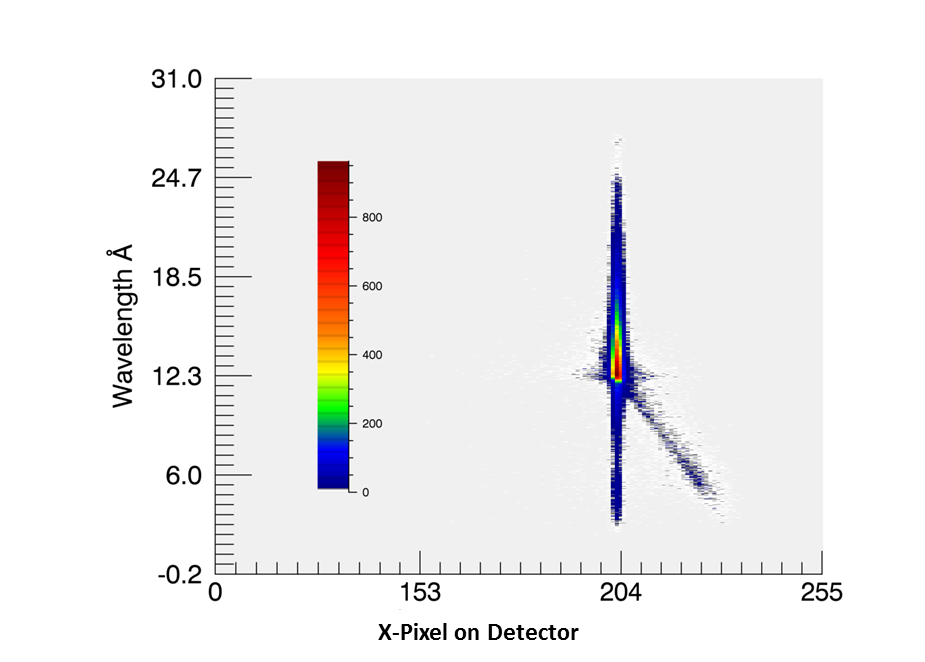


**Spectral Reflection**

**Yoneda Peaks**

**Bragg Scattering**

Figure S4a. D17 2D detector shows evidence of off-specular scattering (scattering of in-plane components), particularly the Yoneda wings. Yoneda scattering occurs at interfaces not perfectly flat – its form is dependent upon the films correlations & scattering power.

.


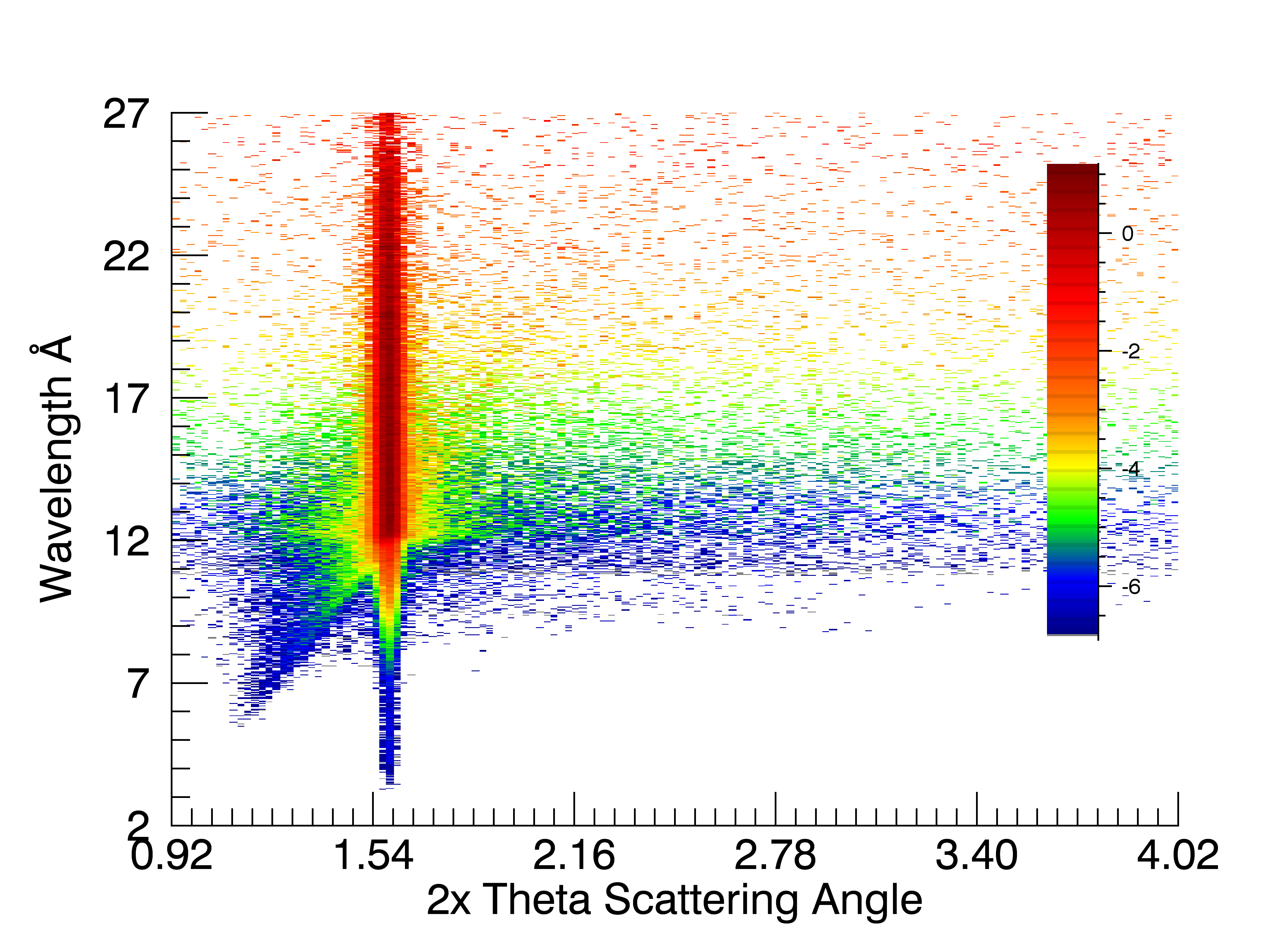


Figure S4b. 2D detector scattering pattern in terms of physical values – wavelength, Å vs. x2 scattering angles of the reflected neutrons. Normalized intensities are represented by a logarithmic color legend.

**Fig. S4b)** 2D detector scattering pattern in terms of real physical values – wavelength, Å vs. 2θ scattering angles of the reflected neutrons. Normalized intensities are represented by a color legend.

1. **Solid-Air Neutron Reflection measurements (Dehydrated Environment)**


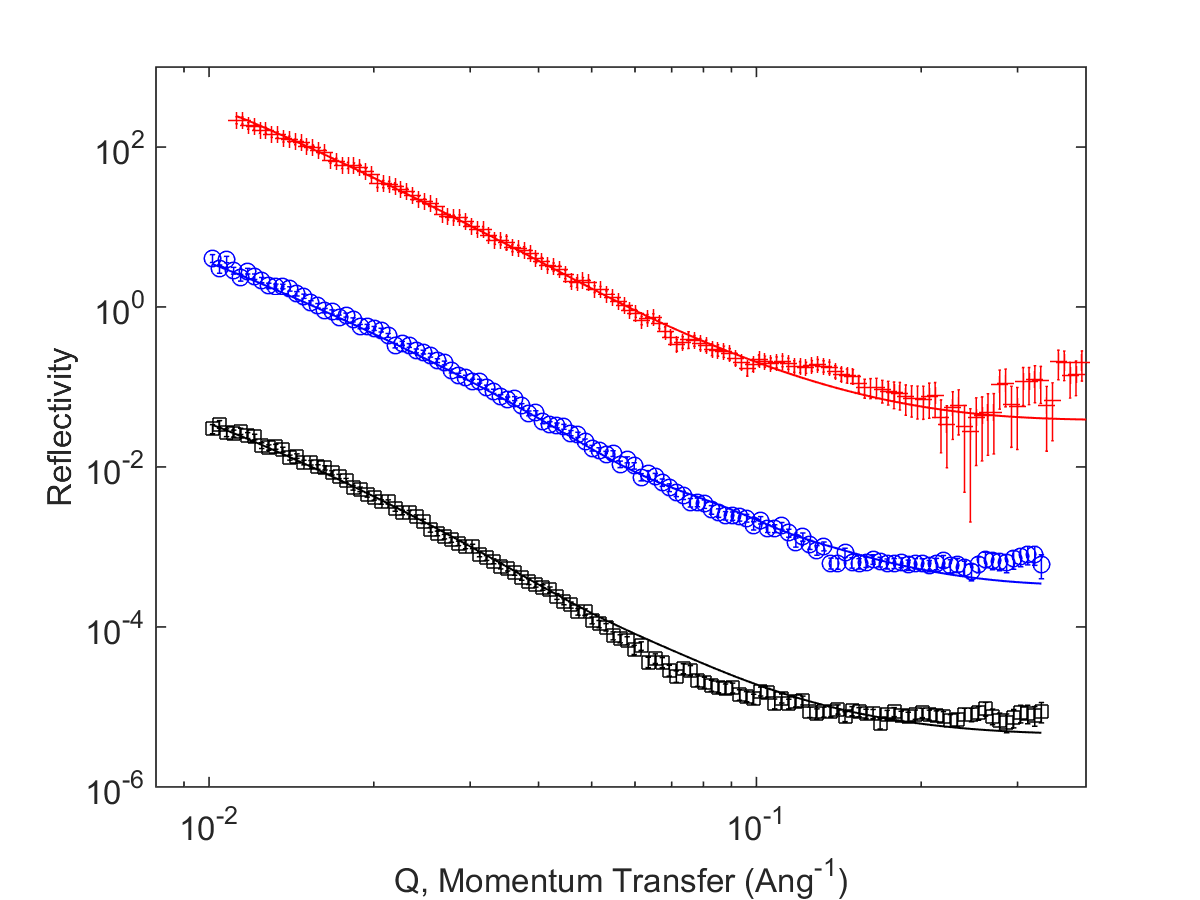

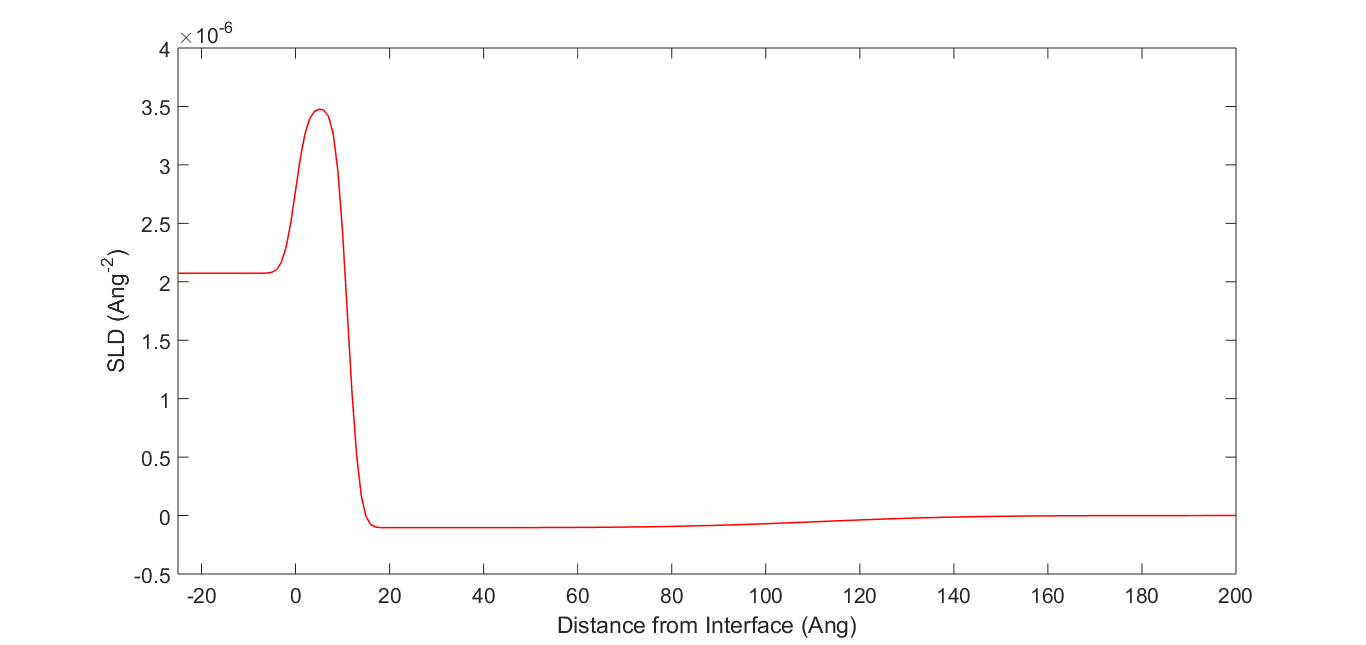


*□* **Dehydrated SCW film**

**O Dehydrated LGW film**

**+ SCW sample – Ambient Conditions**

Figure S5a. Plot shows the solid-air NR plots for the dehydrated SCW and LGW wax films along with a SCW film measured at ambient conditions (normalised reflectivity vs momentum transfer, *Q*). Figure S5b. Shows their associated SLD profiles. All 3 profiles can be fitted to the same model, however due to the poor SLD contrasts, little information can be extracted. Reflection profiles are staggered in multiples of ×100 for clarity.

Table S1: Structural parameters obtained from solid-air NR measurements of dehydrated SCW and LGW model films along with a SCW film measured at ambient conditions. The corresponding NR profiles are shown in Fig. S5a. A Heaviside step function model (described in Section 1.2 – main text) is used to model NR profiles describing the underlying wax film and surface extrusion layer, however little information can be extracted due to the poor SLD contrast.  *****Error bars too large – difficult to distinguish wax substrate from bulk.

| Sample | Wax SLD @ substrate  (X 10^-6^ Å^-2^) | Layer 1 | | | Layer 2 | | Chi-Sq. |
| --- | --- | --- | --- | --- | --- | --- | --- |
|  |  | Thickness, *τ*, (Å) | Heaviside Decay Parameter, *σ*, (Å) | Surface Coverage | Thickness, *τ*, (Å) | Heaviside Decay Parameter, *σ*, (Å) |  |
| Dehydrated Wax Model | -0.3 ± 0.20 | 100* | 30* | - | - | - | 13.97 |

1. **Solid-Air: Fully Hydrated Conditions**

**Incoherent Sum of Reflectivities**


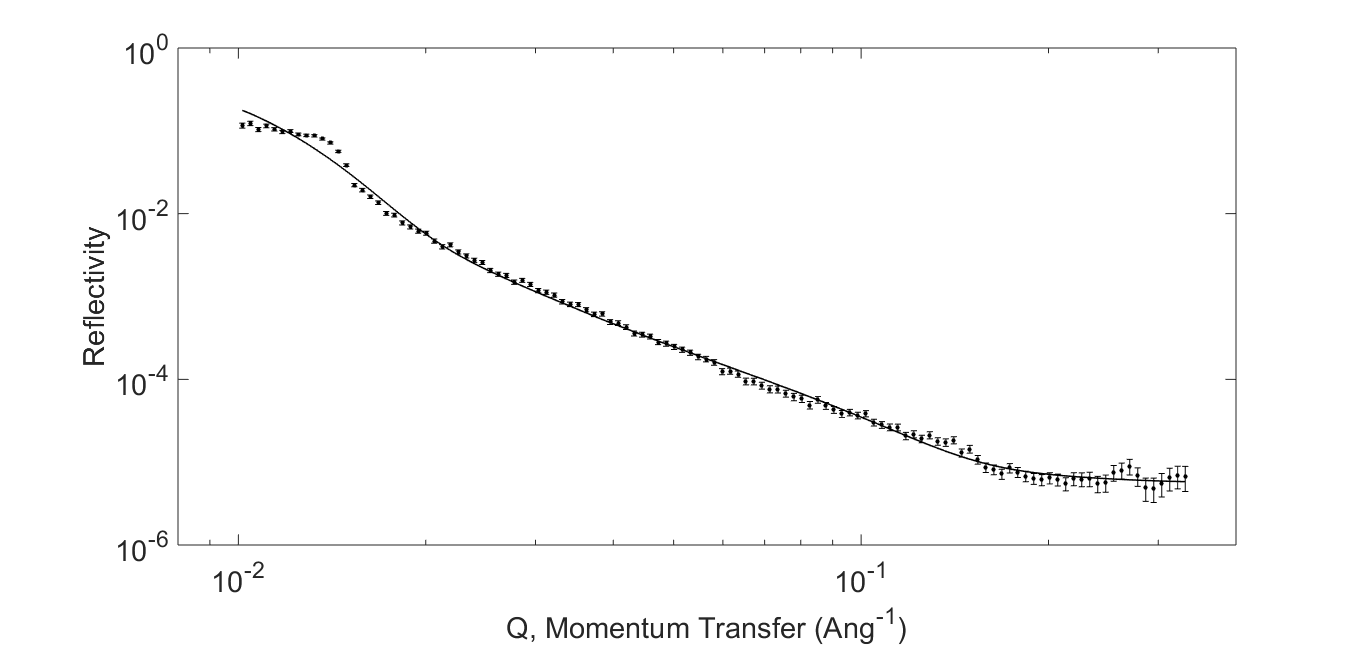

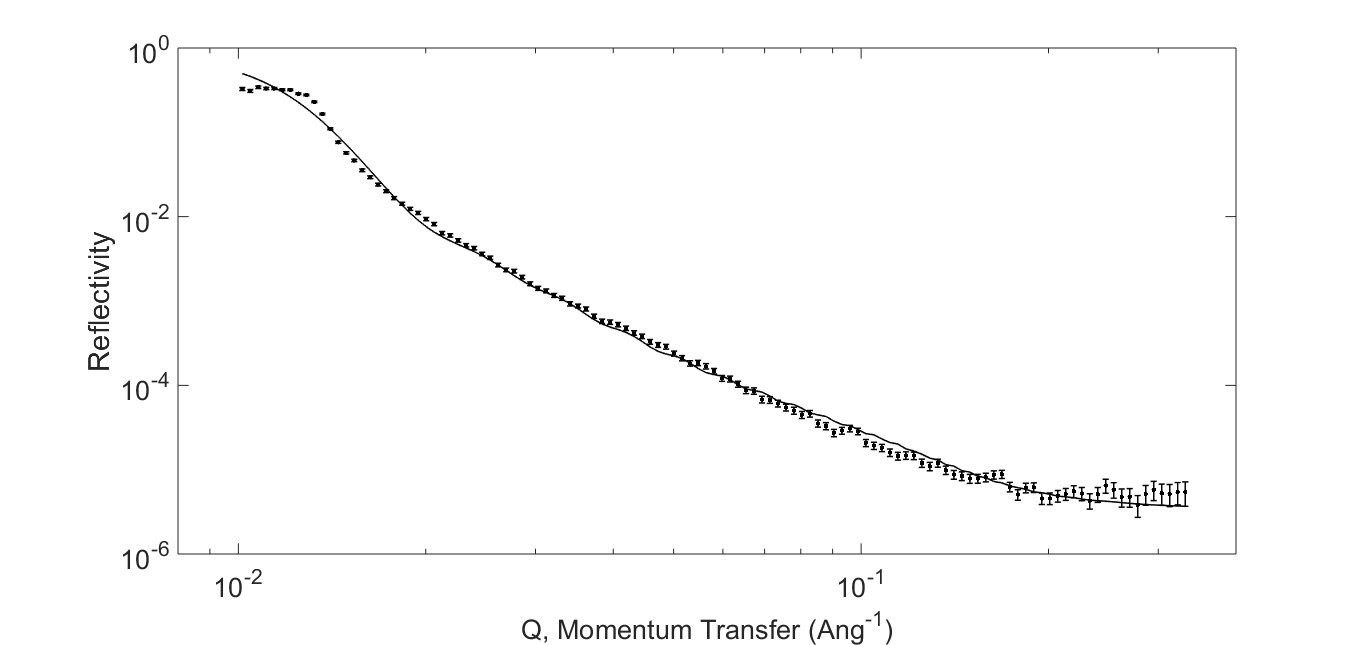


a.) Supercritical CO_2_ extracted waxes (SCW)

b.) Laboratory grown waxes (LGW)

Figure S6. Plots show the incoherently summed solid-air reflectivity profiles for the SCW and LGW wax films measured under saturated humidity conditions. The solid-air and solid-D_2_O components were incoherently added to give the best-fitting final NR profile. By carrying out a scaled sum of reflectivities, the condensed water fraction (which forms the solid-D_2_O component of the NR profile) was found to occupy 10% and 6% of the underlying wax film surface undergoing reflection for the SCW and LGW wax films respectively.

1. **Wheat wax Ellipsometry Model**

**
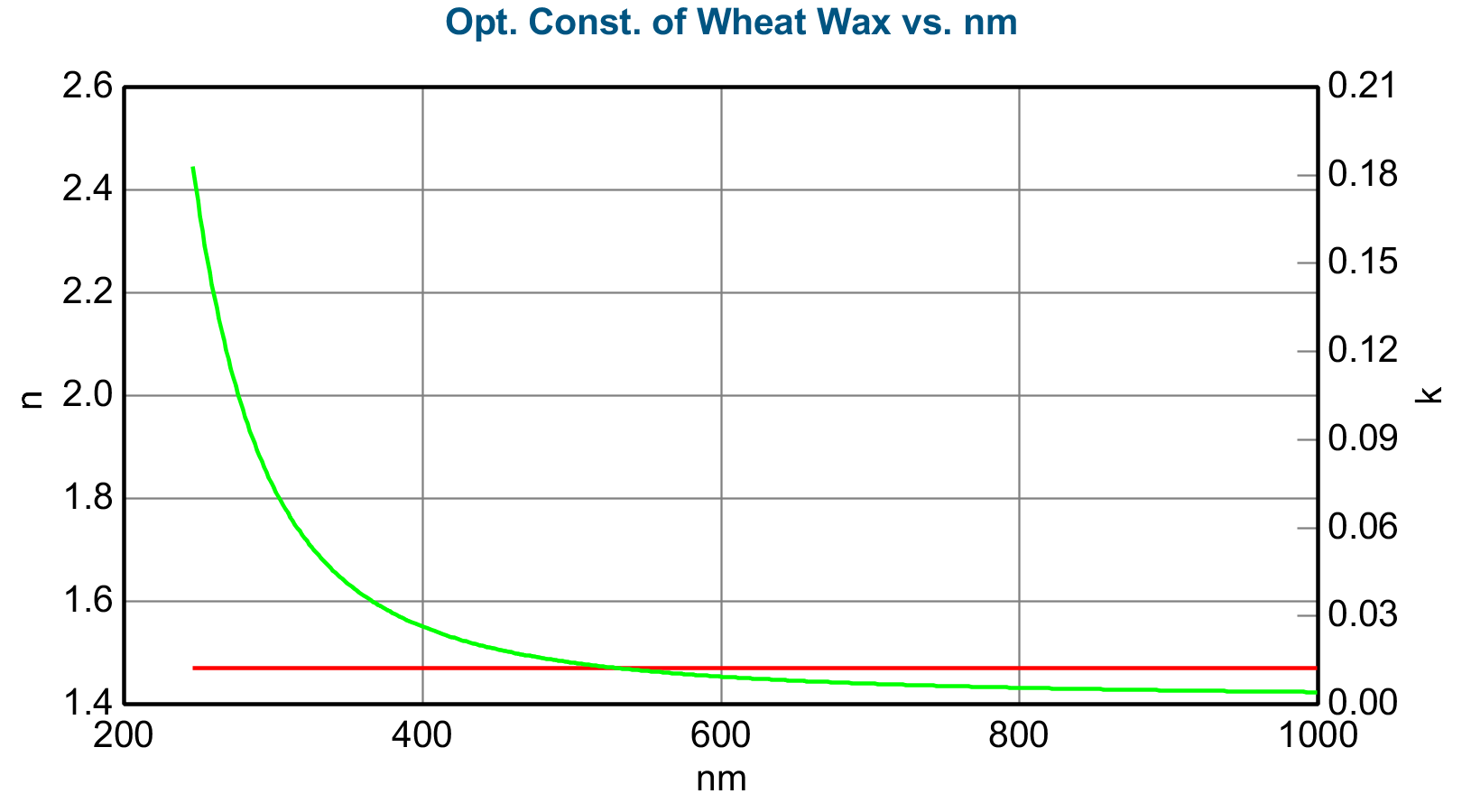
**

Figure S7. Ellipsometry measurements were fit using a user defined wheat wax model; with refractive index, *n*, fixed at 1.47 and an exponentially decaying extinction coefficient, *k(λ)*, with a fixed value of 0.025 at the **sodium** D **line**, 589 nm.

**References**

1. Wang JQ, Jia DH, Tao K, Wang CD, Zhao XB, Yaseen M, et al. Interfacial assembly of lipopeptide surfactants on octyltrimethoxysilane-modified silica surface. Soft Matter. 2013;9(40):9684-91.

2. Alexander S, Eastoe J, Lord AM, Guittard F, Barron AR. Branched Hydrocarbon Low Surface Energy Materials for Superhydrophobic Nanoparticle Derived Surfaces. Acs Appl Mater Inter. 2016;8(1):660-6.

3. Bird JC, Dhiman R, Kwon HM, Varanasi KK. Reducing the contact time of a bouncing drop. Nature. 2013;503(7476):385-+.

4. Shirtcliffe NJ, Aqil S, Evans C, McHale G, Newton MI, Perry CC, et al. The use of high aspect ratio photoresist (SU-8) for super-hydrophobic pattern prototyping. J Micromech Microeng. 2004;14(10):1384-9.
